# Moderating effect of classroom sociable norm on the relations between unsociability and internalizing problems in Chinese adolescents

**DOI:** 10.3389/fpsyt.2023.1168342

**Published:** 2023-06-08

**Authors:** Yihao Hu, Amanda Bullock, Ying Zhou, Junsheng Liu

**Affiliations:** ^1^School of Psychology and Cognitive Science, East China Normal University, Shanghai, China; ^2^Department of Psychology, Carleton University, Ottawa, ON, Canada; ^3^China Executive Leadership Academy Pudong, Pudong, China; ^4^Shanghai Changning Mental Health Center, Shanghai, China

**Keywords:** unsociability, internalizing problems, adolescents, classroom sociable norm, moderating effect

## Abstract

**Objectives:**

The goal of the present study was to examine the moderating effect of classroom sociable norm on the relations between unsociability and internalizing problems (the indicators included depression, loneliness and self-esteem) in Chinese adolescents.

**Methods:**

Participants were *N* = 1,160 adolescents in Grade 4–8 from Shanghai, People’s Republic of China. They completed questionnaires about unsociability, sociability, and social preference via peer nominations, while depression, loneliness, and self-esteem were collected via self-report.

**Results:**

It was found that unsociability was positively associated with depression and loneliness, and negatively associated with self-esteem. Moreover, the relations between unsociability and indicators of internalizing problems were moderated by classroom sociable norm. More specifically, the significant positive associations between unsociability and depression and loneliness were stronger in classrooms with high sociable norm, and the negative association between unsociability and self-esteem was only significant in such classrooms.

**Conclusion:**

The findings suggest that classroom sociable norm plays an important role in unsociable adolescents’ psychological adjustment in China. Researchers should focus more on the influence of classroom environment on adolescents’ development in future.

## Introduction

Unsociability is defined as the non-fearful preference for solitary activities (1). Previous research has postulated that unsociability may be particularly problematic for early adolescence since this is a developmental period in which social norms and expectations about peer interactions are strongly emphasized (2). Indeed, studies have shown that unsociable adolescents experience poor psychosocial adjustment such as internalizing problems (3, 4) and peer difficulties (5–8) as compared to other peers. As such, it is not surprising that researchers have focused their attention on elucidating the factors that could exacerbate or attenuate the relations between unsociability and adolescents’ adjustment (9–11). In the present study, we focused on the role of classroom, an important microsystem for adolescents’ development (12). Specifically, we examined the moderating effect of classroom sociable norm on the relations between unsociability and internalizing problems among Chinese adolescents.

## Links between unsociability and internalizing problems in Chinese adolescents

According to the approach-avoidance motivation model of social withdrawal, unsociable adolescents have both low approach motivation and low avoidance motivation in social situations (13). That is, they do not seek social interactions, but they do not fear or avoid it. Instead, it appears that unsociable individuals prefer to spend time alone (1). In previous research, other terms which had similar meanings to unsociability have also been used, including preference for solitude (14), social disinterest (15) and affinity for aloneness (16).

Although unsociability may be associated with poor adjustment such as loneliness in other countries (17), unsociable adolescents are thought to be at a higher risk for adjustment problems in China than in western countries, given the collectivistic nature of Chinese society that encourages and emphasizes group harmony, cohesion, and interdependence (18). More specifically, adolescents who prefer to spend time alone may be perceived by peers and adults as selfish as their behavior deviates from the social norm in China (19). Indeed, there is a growing body of research that has shown that unsociable Chinese adolescents face internalizing issues such as depression and loneliness (5, 6, 20). For example, a study in China found that unsociability contributed to poorer psychological adjustment (including higher depression, higher loneliness, and lower self-worth) in Chinese children (3). A previous study has found that the group size of unsociable adolescents was not small in China, accounting for about 14.6% of total Chinese adolescents (21), therefore it is important to find protective factors for their psychological adjustment.

## The role of classroom norm on adolescents’ adjustment

Classroom norm refers to either what is commonly done, or what is commonly approved or socially sanctioned in the classroom (22). Specifically, norm salience in the classroom is the extent to which classmates express their attitude towards a type of behavior by virtue of reactions to peers who do it, such as rejection and popularity (23). Compared to descriptive norm or injunctive norm, that is, what most people do or what people are expected to do (23), norm salience of behavior may have a stronger association with peer influence on adolescents’ behavior in the classroom (24).

Previous studies have indicated that norm salience of different behaviors such as bullying (25) and defending (26) in the classroom had an impact on adolescents’ adjustment. For example, it was found that in classrooms where bullying was more rejected, the behavior of defending was more prevalent and popular (25). Moreover, the findings in a previous study indicated that adolescents would have better perceptions of classroom climate and feelings of belonging in classrooms where defending was more popular (26). Therefore, based on these findings, it appears that norm salience may be a key factor in adolescents’ adjustment.

## Moderating role of classroom sociable norm on unsociable adolescents’ internalizing problems

Although there have been studies exploring possible moderators on relations between adolescents’ unsociability and adjustment difficulties, previous researchers have focused more on individual-level variables, such as parenting behavior (11), behavioral control (27), insecure attachment (9) and others’ support (28). According to the goodness of fit theory (29), babies would have better development when their temperament was a good match for their parents’ parenting behavior. As an extrapolation of this theory, we could speculate that individual’s characteristic would be associated with different developmental outcomes in different environments (2). There have been studies indicating that the characteristics of the environment would exert influence on unsociable adolescents’ adjustment, either their groups (30) or their classrooms (31). For example, a study found that solitary play was negatively associated with social preference only in high-interactive groups, that is, where the group members had more social interactions with others (30). Moreover, another study reported that unsociability only positively predicted peer victimization in classrooms with a low prevalence of unsociability (31).

However, the above two research focused on the role of prevalence of behavior, that is, the descriptive norm in the group or classroom. In terms of norm salience, only one study found that norm salience of social withdrawal or aggression moderated the association between social withdrawal and peer victimization (32). According to the reputational salience hypothesis (33), behavior which is popular in a group would become “reputationally salient.” As early adolescence is an important period in which to establish status hierarchy (34), it is possible for adolescents to adopt behaviors which are popular to get status and then acquire material or social resources in the classroom (35). Indeed, there have been studies indicating that adolescents would adopt more behaviors popular in their classrooms for the purpose of improving their status, such as aggressive behavior (36) and prosocial behavior (37).

Previous studies indicated that time spent with peers continues increasing from middle childhood to late adolescence (38), therefore sociability may be more important for adolescents than children. However, there has been no study exploring the effect of norm salience of sociability (hereinafter referred to as “classroom sociable norm”). Based on the findings of previous research (36, 37), it could be speculated that in classrooms with high sociable norm, that is, classrooms in which sociability is more popular, adolescents would tend to have more social interactions with others to acquire popularity for themselves (35). For unsociable adolescents who prefer to participate in solitary activities (1), their behavior may be different from other classmates’ in classrooms with high sociable norm. According to the Theory of Social Comparison (39), people would evaluate their own opinions or abilities in comparisons with others. As a result of comparisons, unsociable adolescents may feel upset and regard themselves as incompetent in classrooms with a high sociable norm and subsequently have more internalizing problems. Moreover, according to the Individual-Group Similarity Model (40), people whose behaviors deviated from the group norms would be rejected by other members in the group. Therefore, unsociable adolescents may be more excluded or rejected in classrooms with high sociable norm, and then their poor peer relationships may contribute to more internalizing problems (3, 41).

## The current study

Previous research has found that the links between unsociability and internalizing problems might be strongest in early adolescence, a period when social norms and expectations about peer interactions are emphasized (2). Indeed, peer interactions are increasingly important from childhood to adolescence (38) and it is important for adolescence to strive for status in their classrooms (34). As a result, exploring the role of classroom sociable norm on adolescents’ adjustment is warranted. Our aim was to explore the moderating effect of classroom sociable norm on relations between adolescents’ unsociability and internalizing problems in China. We chose depression, loneliness, and self-esteem, which were representative indices of psychological adjustment (3) as dependent variables in the current study. Because social preference was highly correlated with popularity in China (42) and it is difficult to find a term in Chinese that directly corresponds to popularity in English (43), we used social preference as the index of status for the calculation of classroom sociable norm.

The following hypotheses were put forward. First, it was hypothesized that Chinese adolescents’ unsociability would be significantly associated with internalizing problems. More specifically, higher unsociability would be associated with higher depression, higher loneliness, and lower self-esteem. Second, it was hypothesized that classroom sociable norm would significantly moderate the relations between unsociability and internalizing problems. More specifically, the relations between unsociability and internalizing problems would be stronger in classrooms with higher sociable norm.

Given that the associations between unsociability and adjustment may be different across gender (44) or developmental stages (2), we included gender and grade as control variables. In addition, previous research has indicated that class size and proportion of boys in the classroom might also influence adolescents’ adjustment (45). As such, we also controlled for the main effect of these two variables.

## Method

### Participants

We recruited participants from four public schools, including two primary schools and two secondary schools in Shanghai, People’s Republic of China. A total of *N* = 1,160 students participated in the study (including 569 boys, *M*_age_ = 11.69 years, *SD* = 1.60 years), including those in Grades 4 (*n* = 264; *M*_age_ = 9.84 years, *SD* = 7.30 months), 5 (*n* = 283; *M*_age_ = 10.83 years, *SD* = 6.80 months), 6 (*n* = 213; *M*_age_ = 11.91 years, *SD* = 9.27 months), 7 (*n* = 179; *M*_age_ = 12.84 years, *SD* = 8.92 months) and 8 (*n* = 221; *M*_age_ = 13.81 years, *SD* = 8.82 months) respectively.

There were 39 classes in total (including 9 classes in Grade 4, 9 classes in Grade 5, 7 classes in Grade 6, 6 classes in Grade 7 and 8 classes in Grade 8), with approximately 30 students in each class on average. Almost 100% of adolescents belonged to Han nationality, the predominant nationality (over 90% of the population) in China (46). All participating students assented to this study and acquired consent from their own parents before data collection. 92% of participants were from intact families, and about 47% of fathers and 42% of mothers had a college or higher education.

### Procedure

The design of the current study was reviewed and approved by the institutional review board of East China Normal University. Written informed consent were obtained from participating students and their parents before data collection. Adolescents with parental consent who agreed to participate were arranged to finish the questionnaire during school hours within their own classrooms. During data collection, each adolescent reported their level of depression, loneliness and self-esteem by self-report measures, and they were also provided a class list to finish peer-nomination assessments, including unsociability, sociability and social preference. The process of data collection was carried out by well-trained graduate research assistants, and the duration of it was about 40 min. In order to protect adolescents from potential negative influence during the data collection, we told them that all of their answers would only be used for research and kept confidential, and they could seek help from our team or psychology teachers in their schools if they needed it. Each participating student received a pen and a notebook as rewards after finishing the questionnaires.

### Measures

#### Unsociability and sociability

Adolescents’ unsociability and sociability were measured using the *Revised Class Play* (RCP) (47, 48) via peer nominations. There are four items for unsociability (e.g., “Someone who prefers playing alone.” “Someone who has no interest in group activities.”) and four items for sociability (e.g., “Someone who has many friends.” “Someone who likes playing with others.”) respectively. Adolescents could nominate up to three classmates on each item. According to previous researcher’s suggestion (49), both same-sex and cross-sex nominations were allowed. For each adolescent, the nominations they received on each item were standardized within classroom and summed for unsociability and sociability respectively, and then the total score was standardized within classroom again. The reliability and validity of this measure have been shown in Chinese adolescents (44). In the current study, the internal consistency of this measure was α = 0.89 for unsociability and α = 0.88 for sociability.

#### Social preference

Following the procedure of previous studies (50), each adolescent was asked to nominate up to three classmates whom they most liked to be with and up to three classmates whom they least like to be with, respectively. Both same-sex and cross-sex nominations were allowed, and then adolescents’ nominations received on each item were standardized within classroom. Social preference was computed by subtracting the standardized score of “like least” from the standardized score of “like most,” and then the total score was standardized within classroom again. This procedure has been demonstrated to be valid in Chinese adolescents (51).

#### Depression

Adolescents’ depression was measured by self-report using the Chinese version of the *Children’s Depression Inventory* (CDI) (52). There were 14 items assessing adolescents’ depressive mood, and each adolescent chose one sentence which best described himself or herself in the past two weeks from the three sentences on each item (e.g., “I am occasionally unhappy.” “I am often unhappy.” “I am usually unhappy.”). The items were answered by using a 3-point scale, with a higher average score of all items indicating a higher level of depression. This measure has been shown to be reliable and valid in Chinese samples (53). In the current study, the internal consistency of this measure was α = 0.85.

#### Loneliness

Adolescents’ loneliness was measured by self-report using the Chinese version of a self-report scale adapted from previous studies (54). It included 16 items (e.g., “I feel lonely.” “It is hard for me to make friends.”), rated on a 5-point scale, with a higher average score of all the items indicating a higher level of loneliness. The reliability and validity of this measure has been demonstrated in Chinese adolescents (20). In the current study, the internal consistency of this measure was α = 0.92.

#### Self-esteem

Adolescents’ self-esteem was measured using a self-report general self-esteem subscale adapted from the *Self-Perception Profile for Children* (SPPC) (55). There were 6 items in total (e.g., “I like myself.” “I am very confident in myself.”) and adolescents responded to them on a 5-point scale, with higher average score of all items indicating higher level of self-esteem. This measure has been shown to be reliable and valid in Chinese adolescents (56). In the current study, the internal consistency of this measure was α = 0.82.

#### Classroom sociable norm

Based on the definition of norm salience (23) and procedure in previous research (32), the classroom sociable norm for each class was acquired by calculating the Pearson correlation coefficient between the scores of sociability and social preference within the classroom. A higher value of classroom sociable norm indicated that sociability was more preferred in the classroom.

### Data analysis

Data analysis was conducted using IBM SPSS for Windows (version 25) and *Mplus* version 7.4 (57). To test the hypotheses, we analyzed the data collected in the current study in the following steps. To begin with, for the descriptive statistics, the correlations among individual-level variables, including unsociability, sociability, social preference, depression, loneliness and self-esteem, and among classroom-level variables, including classroom sociable norm, class size, and proportion of boys in the classroom were calculated. Moreover, multivariate analysis of variance (MANOVA) and t-tests of individual-level variables on gender (boys = 0, girls = 1) and grade (primary school = 0, secondary school = 1) were also conducted.

Next, the multilevel models were employed to examine the moderating effect of classroom sociable norm. The unconditional models, the individual-level models and the classroom-level models for depression, loneliness and self-esteem were examined in *Mplus* 7.4, respectively. First, the dependent variables were included in the unconditional models to examine the between-group variation of them. Second, unsociability, gender, and the interaction of them were added into the individual-level models, to examine the main effect of unsociability on internalizing problems. Third, all classroom-level variables and the interactions of unsociability-grade and unsociability-classroom sociable norm were added into classroom-level models, to examine the moderating effect of classroom sociable norm. Simple slope test was conducted if any significant moderating effect was found (58). Gender and all classroom-level variables were grand-mean centered in the data analysis. Missing data were handled using the full information maximum likelihood method (59) with the MLR estimation in *Mplus* 7.4. The equation of the models is presented below (i = student, j = classroom, e and u were random effects).

Individual-level:


$$\begin{array}{l} \mathrm{Depression}/\mathrm{Loneliness}/\mathrm{Self}-\mathrm{esteem}=\beta_{\mathrm{0j}}+\beta_{\mathrm{1j}}{}^{*}\mathrm{Unsociability}+\\ \beta_{\mathrm{2j}}{}^{*}\mathrm{Gender}+\beta_{\mathrm{3j}}{}^{*}\left({\mathrm{Unsociability}}^{*}\mathrm{Gender}\right)+e_{\mathrm{ij}} \end{array}$$

Classroom-level:


$$\begin{array}{l} \beta_{\mathrm{0j}}=\gamma_{00}+\gamma_{01}{}^{*}\mathrm{Classroom sociable}\;norm+\gamma_{02}{}^{*}\mathrm{Grade}+\\ \gamma_{02}{}^{*}\mathrm{Class size}+\gamma_{03}{}^{*}\mathrm{Proportion of boys}+u_{\mathrm{0j}} \end{array}$$

$$\beta_{\mathrm{1j}}=\gamma_{10}+\gamma_{11}{}^{*}\mathrm{Classroom sociable}\;norm+\gamma_{12}{}^{*}\mathrm{Grade}+u_{\mathrm{1j}}$$

## Results

### Missing data

There was no missing data on variables acquired via peer nominations, but on depression, loneliness and self-esteem, which were self-reported, the percentage of missing data was all 2.2%. The result of Little’s MCAR test (60) indicated that *χ*^2^ (2169) = 2960.29, *p* < 0.001, which meant that data were not missing at random. However, according to the criterion stipulated in previous research (61), *χ*^2^/df = 1.36 < 2, it was surmised that the data were missing completely at a random pattern.

### Descriptive statistics

Means and standard deviations for, and intercorrelations among individual-level or classroom-level study variables are shown in Table 1. For individual-level variables, there were significant positive correlations between unsociability and depression and loneliness, and significant negative correlations between unsociability and sociability, social preference, and self-esteem. For classroom-level variables, there was no significant correlation between each other.

**Table 1:** Descriptive statistics of study variables.

	1	2	3	4	5	6
Individual-level variables						
1 Unsociability						
2 Sociability	−0.17^***^					
3 Social preference	−0.33^***^	0.41^***^				
4 Depression	0.22^***^	−0.20^***^	−0.27^***^			
5 Loneliness	0.30^***^	−0.26^***^	−0.33^***^	0.69^***^		
6 Self-esteem	−0.12^***^	0.14^***^	0.16^***^	−0.58^***^	−0.53^***^	
*M*	0.02	0.03	0.00	1.41	1.92	3.44
*SD*	1.00	0.99	0.98	0.34	0.73	0.81
Classroom-level variables						
1 Classroom sociable norm						
2 Class size	−0.17					
3 Proportion of boys	−0.15	−0.06				
*M*	0.42	29.74	0.49			
*SD*	0.16	3.91	0.06			

^***^p < 0.001.

The results of the MANOVA revealed that the main effect of gender was significant, Wilk’s λ = 0.96, *F* (6, 1,124) = 7.85, *p* < 0.001, η^2^ = 0.04; the main effect of grade was significant, Wilk’s λ = 0.92, *F* (6, 1,124) = 15.46, *p* < 0.001, η^2^ = 0.08; and the interactive effect of gender-grade was significant, Wilk’s λ = 0.98, *F* (6, 1,124) = 15.46, *p* < 0.01, η^2^ = 0.02. In the follow-up analysis of variance, gender had a significant effect on sociability (*F* (1, 1,156) = 10.86, *p* < 0.01), social preference (*F* (1, 1,156) = 40.48, *p* < 0.001), depression (*F* (1, 1,131) = 12.38, *p* < 0.001) and loneliness (*F* (1, 1,131) = 13.36, *p* < 0.001). Grade had a significant effect on depression (*F* (1, 1,131) = 48.42, *p* < 0.001), loneliness (*F* (1, 1,131) = 60.78, *p* < 0.001) and self-esteem (*F* (1, 1,131) = 43.06, *p* < 0.001). There were significant interaction effects of gender and grade on depression (*F* (1, 1,131) = 5.32, *p* < 0.05) and self-esteem (*F* (1, 1,131) = 12.71, *p* < 0.001). The means and standard deviations for individual-level study variables for different gender and grade are shown in Table 2.

**Table 2:** The means and standard deviations of individual-level variables (*M* ± SD).

	Primary school		Secondary school	
	Boys	Girls	Boys	Girls
Unsociability	0.08 ± 0.96	−0.05 ± 1.03	0.01 ± 0.92	0.04 ± 1.09
Sociability	−0.15 ± 0.81	0.13 ± 1.09	0.00 ± 0.97	0.11 ± 1.03
Social preference	−0.25 ± 1.06	0.22 ± 0.86	−0.12 ± 1.00	0.13 ± 0.95
Depression	1.40 ± 0.34	1.28 ± 0.29	1.49 ± 0.35	1.47 ± 0.35
Loneliness	1.85 ± 0.73	1.65 ± 0.66	2.14 ± 0.74	2.02 ± 0.70
Self-esteem	3.48 ± 0.82	3.72 ± 0.76	3.34 ± 0.83	3.24 ± 0.76

### Multilevel model

#### The unconditional models

The intraclass correlations (ICC) of depression, loneliness, and self-esteem were 0.085, 0.078 and 0.081, respectively. According to the ICCs and class size, the design effect of depression, loneliness, and self-esteem were 3.47, 3.26, and 3.35 respectively, all of which were above 2.0, indicating that there was a need for multilevel modeling in the data analysis (62).

#### The individual-level models

Random slope models for depression, loneliness, and self-esteem were all examined. As shown in Table 3, unsociability had significant positive relations with depression (*b* = 0.074, *SE* = 0.011, *p* < 0.001) and loneliness (*b* = 0.218, *SE* = 0.026, *p* < 0.001), and a significant negative relation with self-esteem (*b* = −0.096, *SE* = 0.027, *p* < 0.001) after controlling for gender and the interaction of unsociability and gender.

**Table 3:** The individual-level models of unsociability on internalizing problems.

	Depression		Loneliness		Self-esteem	
	*b*	*SE*	*b*	*SE*	*b*	*SE*
Intercept	1.409^***^	0.019	1.919^***^	0.039	3.441^***^	0.043
Unsociability	0.074^***^	0.011	0.218^***^	0.026	−0.096^***^	0.027
Gender	−0.067^**^	0.021	−0.155^***^	0.043	0.066	0.052
Unsociability × Gender	−0.021	0.025	−0.014	0.051	−0.027	0.052
*Random effect*						
Residual	0.099^***^	0.006	0.428^***^	0.024	0.585^***^	0.033
Intercept	0.010^***^	0.003	0.043^***^	0.012	0.054^**^	0.016
Slope	0.001	0.001	0.011^*^	0.005	0.008	0.007

^*^p < 0.05; ^**^p < 0.01; ^***^p < 0.001.

#### The classroom-level models

**Figure 1:**
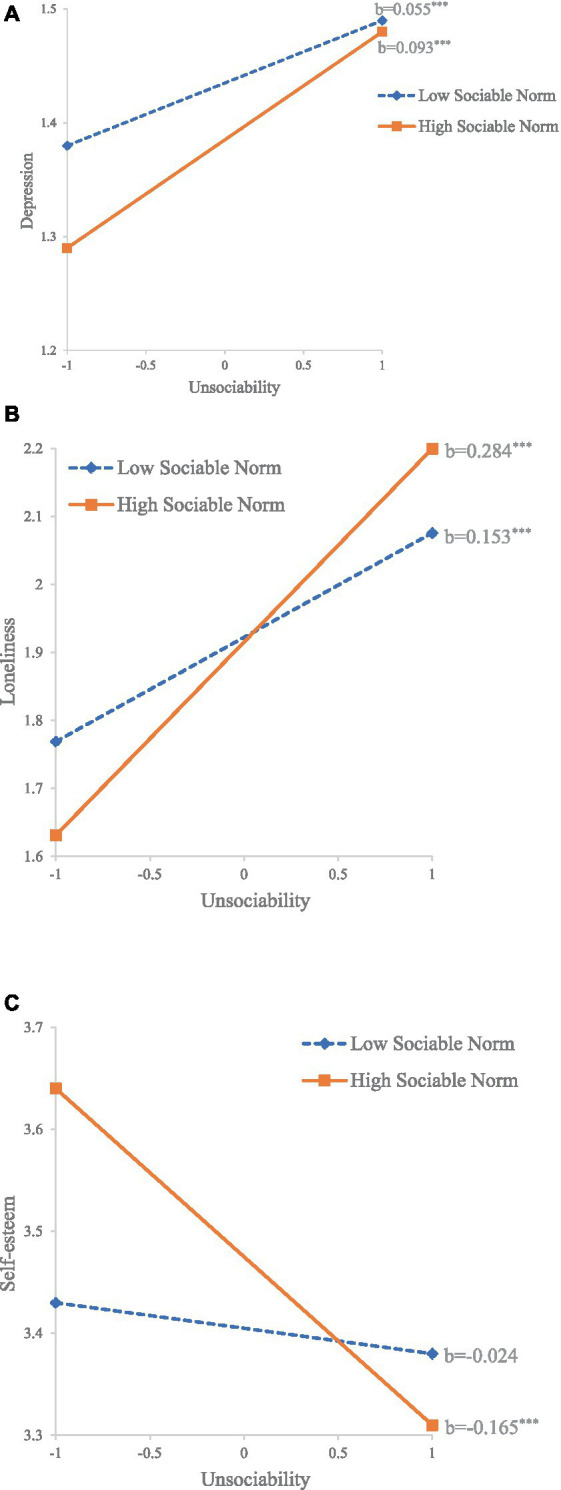
The moderating effect of classroom sociable norm on the relationships between unsociability and internalizing problems: **(A)** Depression; **(B)** Loneliness; **(C)** Self-esteem. ^*^p < 0.05; ^**^p < 0.01; ^***^p < 0.001.

Classroom-level models for depression, loneliness and self-esteem were examined, respectively. As shown in Table 4, for depression, it had a trend of negative relation with classroom sociable norm (*b* = −0.157, *SE* = 0.089, *p* = 0.078). There was a trend of moderating effect of classroom sociable norm on the association between unsociability and depression (*b* = 0.118, *SE* = 0.064, *p* = 0.063), and the simple slope test (see Figure 1A) showed that unsociability had a more positive relation with depression in classrooms with a high sociable norm (z score of classroom sociable norm = +1) (*b* = 0.093, *SE* = 0.015, *p* < 0.001) than in classrooms with a low sociable norm (z score of classroom sociable norm = −1) (*b* = 0.055, *SE* = 0.014, *p* < 0.001). As for loneliness, there was a trend of moderating effect of grade on the association between unsociability and it (*b* = 0.087, *SE* = 0.044, *p* = 0.050), and the simple slope test showed that unsociability had a more positive relation with loneliness in secondary schools (*b* = 0.258, *SE* = 0.029, *p* < 0.001) than in primary schools (*b* = 0.172, *SE* = 0.035, *p* < 0.001). The interaction of unsociability and classroom sociable norm on loneliness was also significant (*b* = 0.413, *SE* = 0.202, *p* = 0.041), and the simple slope test (see Figure 1B) showed that unsociability had a more positive relation with loneliness in classrooms with a high sociable norm (*b* = 0.284, *SE* = 0.041, *p* < 0.001) than in classrooms with a low sociable norm (*b* = 0.153, *SE* = 0.038, *p* < 0.001). As for self-esteem, the interaction of unsociability and classroom sociable norm was also significant (*b* = −0.445, *SE* = 0.176, *p* = 0.012), and the simple slope test (see Figure 1C) showed that unsociability had a significant negative relation with self-esteem in classrooms with a high sociable norm (*b* = −0.165, *SE* = 0.036, *p* < 0.001) but not in classrooms with a low sociable norm (*b* = −0.024, *SE* = 0.038, *ns*).

**Table 4:** The classroom-level models of unsociability on internalizing problems.

	Depression		Loneliness		Self-esteem	
	*b*	*SE*	*b*	*SE*	*b*	*SE*
Intercept	1.410^***^	0.014	1.919^***^	0.027	3.440^***^	0.034
*Individual-level*						
Unsociability	0.074^***^	0.011	0.219^***^	0.023	−0.094^***^	0.024
Gender	−0.063^**^	0.021	−0.147^**^	0.044	0.054	0.053
Unsociability × Gender	−0.019	0.024	−0.003	0.048	−0.047	0.058
*Classroom-level*						
Grade	0.135^***^	0.030	0.378^***^	0.058	−0.323	0.074
Class size	0.001	0.004	0.009	0.008	−0.004	0.009
Proportion of boys	0.016	0.328	−0.746	0.534	0.317	0.688
Classroom sociable norm	−0.157^†^	0.089	−0.023	0.167	0.208	0.256
*Cross-level*						
Unsociability × Grade	0.012	0.020	0.087^†^	0.044	−0.013	0.047
Unsociability × Norm	0.118^†^	0.064	0.413^*^	0.202	−0.445^*^	0.176
*Random effect*						
Residual	0.099^***^	0.006	0.426^***^	0.024	0.585^***^	0.033
Intercept	0.004^**^	0.001	0.016^*^	0.007	0.028^*^	0.011
Slope	0.001	0.001	0.008	0.005	0.003	0.005

^†^*p* < 0.10; ^*^p < 0.05; ^**^p < 0.01; ^***^p < 0.001.

## Discussion

The main goal of current study was to examine the moderating effect of classroom sociable norm on the relations between unsociability and internalizing problems among Chinese adolescents. Our hypotheses were supported by the results. Unsociability was associated with higher levels of depression and loneliness as well as a lower level of self-esteem. In addition, the analyses of multilevel models indicated that the effects of unsociability on internalizing problems were stronger in classrooms with a high sociable norm. More specifically, the significant positive associations between unsociability and depression and loneliness were stronger in classrooms with a high sociable norm, and the negative association between unsociability and self-esteem was only significant in classrooms with a high sociable norm.

Regarding the relations between unsociability and indicators of internalizing problems, the results in current study were consistent with those from previous research conducted in contemporary Chinese societies (5, 44). More specifically, unsociability was significantly and positively related to depression and loneliness, but significantly and negatively related to self-esteem in the current sample. Influenced by the traditional Chinese culture, getting along well with others has been emphasized in China for thousands of years (18), even in Shanghai, which is a large modern city of China. There were two possible mechanisms explaining the negative influence of unsociability on adolescents’ mental health. To begin with, spending time alone may be viewed as selfish and indifferent under the background of collectivism (19). Therefore unsociable adolescents would likely be rejected or disliked by their peers, contributing to their internalizing problems (3). Additionally, because unsociable adolescents prefer to spend time alone (63), they may have concerns about their own social competence and have lower self-efficacy on social interactions, which would lead to later internalizing problems.

For the moderating effect of classroom sociable norm, we found that the associations between unsociability and internalizing problems were stronger in classrooms with a high sociable norm. More specifically, unsociable adolescents were more likely to have higher levels of depression or loneliness, or a lower level of self-esteem in classrooms where sociability was more preferred. Based on the reputational salience hypothesis (33) and the theory of resource control (35), social behavior would allow one to earn a reputation for oneself in classrooms with a high sociable norm, and students may tend to adopt it in order to enhance their own status in the classroom. From this perspective, unsociable adolescents may feel incompatible with the whole classroom climate since they prefer participating in solitary activities (1). Moreover, according to the Optimal Distinctiveness Model (64), individuals have both needs for assimilation and differentiation from others in the group. Therefore, unsociable adolescents would feel that they were too unique in classrooms with a high sociable norm and their need for assimilation would not be satisfied. As a consequence, they may have been deprived of a sense of belonging which may explain why they would feel lonely in such a classroom.

Furthermore, according to the Individual-Group Similarity Model (40), other students in classrooms with a high sociable norm may reject or even victimize adolescents who displayed unsociable behavior (31, 32). However, because everyone has interpersonal needs such as inclusion, affection and control (65), it could be speculated that unsociable adolescents would feel upset in classrooms with high sociable norm since their interpersonal needs were not met from their classmates. This in turn, could result in emotional disturbance to develop such as depressed mood. Moreover, they might suspect that their self-worth was low after they lost a sense of control in their interpersonal relationships, which might explain why they had a lower level of self-esteem in such a classroom climate.

Taken together, the present study is the first to provide insight on the moderating role of classroom sociable norm in the relations between adolescents’ unsociability and internalizing problems. Nevertheless, there are several limitations that could be addressed in future research. First, our study was cross-sectional in nature, thereby making it challenging to draw causal inferences about the direction of effects that were found. Therefore, it is recommended that future researchers conduct longitudinal studies to explore the timing effect further. Second, unsociability is characterized as both low motivation of approach and low motivation of avoidance (13), but it was measured using peer nominations in the current study. Therefore, it could be questioned whether the measure of unsociability accurately reflected the internal state of adolescents. Researchers could measure unsociability by self-report in the future, which might reveal participants’ motivations better. Third, our sample was from Shanghai, a modern city in China. Future researchers could consider exploring the moderating effect of classroom sociable norm in rural areas of China where collectivism may be more encouraged (5). Fourth, we did not explore which factors could mediate the associations between unsociability and internalizing problems while examining the moderating effect of classroom sociable norm. Possible mediators such as peer relations (3) could be explored in future research.

In spite of the above limitations, there are still some meaningful implications from our study. For the theoretical implications, the findings in the current study demonstrated that classroom sociable norm had an influence on relations between unsociability and internalizing problems for Chinese adolescents. Therefore, the important role of classroom environment, especially for unsociable adolescents was underscored. It is recommended that future researchers explore if other classroom-level variables, such as classroom status hierarchy (34) and classroom aggressive norm (24) could influence unsociable adolescents’ adjustment. For the practical implications, the findings from this study could enlighten educators in school that classroom environment is vital for students’ development and that sociability is an important characteristic in school life. Therefore, educators could try to establish a benign classroom climate for students, such as telling them to be kind to classmates irrespective of how sociable they are. Moreover, educators should cultivate unsociable adolescents’ social competence with some measures, such as teaching them proper social skills and encouraging them to have interactions with other students.

## Conclusion

This study was aimed at exploring the moderating effect of classroom sociable norm on the relations between unsociability and internalizing problems in Chinese adolescents. Consistent with our hypotheses, unsociability was associated with more internalizing problems, including higher levels of depression and loneliness, as well as lower level of self-esteem in Chinese adolescents. Moreover, these associations were stronger in classrooms with a higher classroom sociable norm, that is, classrooms where sociability was more preferred by students. The results indicated that unsociable adolescents’ adjustment would be influenced by the classroom environment in China and it is essential to explore more classroom-level protective or risk factors for them in the future.

## Data availability statement

The raw data supporting the conclusions of this article will be made available by the authors, without undue reservation.

## Ethics statement

The studies involving human participants were reviewed and approved by Institutional Review Board of East China Normal University. Written informed consent to participate in this study was provided by the participants’ legal guardian/next of kin.

## Author contributions

YH and JL contributed to conception and design of the study. JL organized the database. YH performed the statistical analysis and wrote the first draft of the manuscript. YH, AB, YZ, and JL wrote sections of the manuscript. All authors contributed to the article and approved the submitted version.

## Conflict of interest

The authors declare that the research was conducted in the absence of any commercial or financial relationships that could be construed as a potential conflict of interest.

## Publisher’s note

All claims expressed in this article are solely those of the authors and do not necessarily represent those of their affiliated organizations, or those of the publisher, the editors and the reviewers. Any product that may be evaluated in this article, or claim that may be made by its manufacturer, is not guaranteed or endorsed by the publisher.
